# T677T Methylenetetrahydrofolate Reductase Single Nucleotide Polymorphisms Increased Prevalence in a Subgroup of Infertile Patients with Endometriosis

**DOI:** 10.1089/jwh.2022.0019

**Published:** 2022-10-26

**Authors:** Patrice Clément, Silvia Alvarez, Laetitia Jacquesson-Fournols, Dominique Cornet, Arthur Clément, Michel Brack, Marc Lalau-Keraly, Didier Delafontaine, Marc Cohen, Yves Menezo

**Affiliations:** ^1^ Laboratoire CLEMENT, Department of Genetics and Assisted Reproduction, Paris, France.; ^2^ Cabinet Médical, Gynecologie et Obstetrique, Paris, France.; ^3^ Cabinet Médical Endocrinologie, Paris, France.; ^4^ The Oxidative Stress College, Paris, France.; ^5^ Clinique Natecia Lyon, Gynecologie et Obstetrique, Lyon, France.

**Keywords:** endometriosis, hypofertility, MTHFR SNPs, c.677C>T, c.1298A>C, methylation, oxidative stress

## Abstract

**Background:**

Approximately 10% (190 million) of women worldwide are affected by endometriosis, ectopic deposits of endometrial tissue that create a major source of pain that affects lifestyle and reproductive function. The pathogenesis of endometriosis is an estrogen-dependent inflammatory process, influenced/catalyzed by oxidative stress and consequently defective methylation, with biochemical features centered around the folate and one-carbon cycles. We aimed to determine whether a link could be found between the two major methylenetetrahydrofolate reductase single nucleotide polymorphisms (MTHFR SNPs), c.677C>T and c.1298A>C, involved in methylation process/epigenetic marking failures, and endometriosis.

**Material and Methods:**

We studied a population of 158 patients in a group of >1500 referred for treatment of infertility. All the patients had experienced >2 failed assisted reproductive technology cycles and/or >2 miscarriages, a classical cohort for investigation in our group. Patients with endometriosis had at least stage 2+ disease confirmed by laparoscopy.

**Results:**

The prevalence of the homozygous c.677C>T isoform is doubled in the endometriosis group, 21.5% versus 10.2% in the non-endometriosis group (*p* > 0.01). Symmetrically, the percentage of patients in the endometriosis group with the wild type MTHFR significantly decreased by one-half (8.2%–17.2%) in the non-endometriosis group (*p* < 0.001).

**Conclusion:**

Determination of MTHFR c.677C>T should not be overlooked in patients with harmful endometriosis affecting their fertility. As folates metabolism is impaired in these MTHFR SNPs carrier patients, co-treatment with 5-methyl folate may constitute a successful (co)-treatment modality.

## Introduction

Endometriosis is an estrogen-dependent inflammatory process contributing to subfertility that affects 1 out of 10 women worldwide. Oxidative stress is a major component of the disease^1–3^; antioxidant defenses such as superoxide dismutase and glutathione are overflown. Independently of the specific generation of biochemical hazards, reactive oxygen species contribute to errors in methylation/epigenetic modifications,^4^ and endometriosis does not escape this hazard.^5–7^

**FIG. 1.**
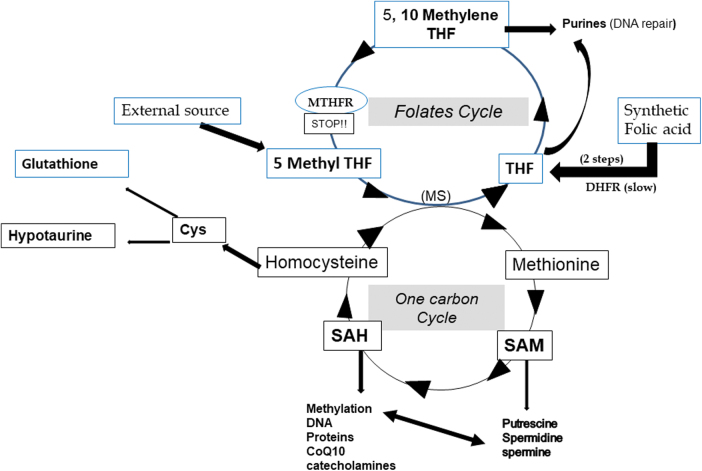
Interactions/cooperation between the one-carbon cycle and the folate cycle for methylation and synthesis of antioxidants. Methylation steps are necessary for the synthesis of CoQ10 (3 steps for ubiquinone synthesis). MTHFR SNPs reduce the capacity (up to 75%) for synthetizing 5-MTHF, the active compound for recycling homocysteine in methionine. The impact on the synthesis of polyamines regulating the methylation process: Low homocysteine recycling leads to poor re-formation of SAM and de-regulates methylation. The DHFR is slow in human. DHFR, dihydrofolate reductase; MS, methionine synthase; 5-MTHF, 5 methyl tetrahydrofolate; MTHFR, methylenetetrahydrofolate reductase; SAH, S-adenosylhomocysteine; SAM, S-adenosyl methionine; SNPs, single nucleotide polymorphisms; THF, tetrahydrofolate.

Excess of estradiol feeds oxidative stress *via* the estrogen receptors signaling: it impairs the epigenetically mediated regulation of gene expression.^7,8^ Methylenetetrahydrofolate reductase (MTHFR) and homocysteine recycling are at the epicenter of antioxidant defense and epigenesis. Elevation in circulating homocysteine metabolism is a cause and a consequence of oxidative stress.^9^ Methylation processes and prevention of oxidative stress have the one-carbon and folate cycles in common (Fig. 1).^4,10^ Scientific literature and biochemical evidence link commonly the one-carbon cycle and the folate status to oxidative stress.

MTHFR plays a central role in these cycles, responsible for production of the active form of folate required for homocysteine re-methylation to methionine. The two principal MTHFR single nucleotide polymorphism (SNP) variants, c.677C>T, also called C677T(Ala222Val) and c.1298A>C, also called A1298C(Glu429Ala) reduce the activity of the enzyme, with an impact on both of these metabolic cycles that results in detrimental effects on methylation processes and an increase in circulating homocysteine.

Formation of 5 methyl tetrahydrofolate (5-MTHF), the active substrate for methionine synthase (MS) can be impaired by up to 70% in the presence of the homozygous c.677C>T isoform. In c.1298A>C homozygotes, MS enzymatic activity may reach only 68% of normal. Alteration of the MTHFR activity is not observed in the wild-type (WT) patients: they do not carry one of these two isoforms. Compound heterozygous mutation can also substantially affect gene activity^11–13^ and the regeneration of homocysteine *via* the 1-CC, and thus affects the process of methylation.

In addition, this lack of methionine regeneration and the resulting impairment of S-adenosyl methionine formation alters the formation of polyamines, active regulators/controllers of methylation in conjunction with the one-carbon cycle (Fig. 1; Soda^14^). As methylation dysregulation and oxidative stress are heavily involved in endometriosis, we wanted to evaluate the prevalence of the two main MTHFR SNPs, c.677C>T and c.1298A>C, in a subpopulation of patients with stage 2+ confirmed endometriosis in a cohort of couples consulting for a history of long-standing infertility^15^ of multiple etiologies. These two SNPs are known to affect both male and female fertility, as well as embryonic development^16–20^ confirming the major role of methylation in reproductive physiology.^21^

## Materials and Methods

### Ethical considerations

Determination of the two SNPs is a standard procedure in our infertility units, subject to the classical rules applied to all genetic tests in a certified laboratory. No human study approval (institutional review board) from Agence de Biomedicine is required, but testing must be prescribed by an andrologist, gynecologist/obstetrician, or an endocrinologist, in patients seeking fertility treatments. Patient informed consent is also required to allow anonymous publication of the data.

This test is classically recommended in our practice for patients suffering infertility of at least 2 years' duration with failed assisted reproductive technology attempts and/or at least two miscarriages; the women should have no specific shifts in the analyses recommended by the ESHRE consensus and the male partner should undergo sperm DNA fragmentation and decondensation tests. The analysis is not reimbursed by social security. A diagnosis of endometriosis is determined in three steps: (1) description of the classical symptoms (pain, metrorrhagia, etc.) during the first interview with the clinician, (2) echography and MRI scan if the suspicion is reinforced, and (3) final confirmation by laparoscopy and eventual cytology.

### Determination of the 2 MTHFR SNPs

The protocol has been previously described.^13,22^ The loop-mediated isothermal amplification human MTHFR mutation kit based on a hybridization technique was used, which requires a 5-μL blood sample. Amplification is performed at 65°C, using several sets of primers simultaneously. Six specific primers covering the locus of the mutation are used for each SNP. Two loop primers are used in both, and the probes used simultaneously amplify the WT gene. The results are obtained by comparison of the fluorescence curves.

### Population

It is a retrospective study concerning an overall population of 1588 women with a variety of different infertility etiologies (including repeated miscarriages) tested for the two SNPs, during the time period from mid-2019 to end of 2020. In this population, only 158 had stage 2+ over endometriosis, according to the American Society for Reproductive Medicine (ASRM) consensus^23^ (mean age 37.6 years, range 32–44 years). Patients with endometriosis had at least stage 2+ (confirmed by laparoscopy) disease. The incidence of c.677C>T and c.1298A>C SNPs in this endometriosis group was compared with the remaining 1430 women in the cohort.

### Statistical analysis

Statistical analysis of the distribution (percentages, Table 1) was performed according to Pearson, likelihood ratio (Wilks), and Fisher's exact tests and confirmed by the adjusted Wald test. The odd ratios were also calculated (Table 2).

**Table 1:** Statistical Analysis of the Frequency Differences in Methylenetetrahydrofolate Reductase Single Nucleotide Polymorphisms Observed in Endometriosis Patients and the General Hypofertile Population

C677c>T SNP configuration	c.677C>T	c.677C>T	c.677C>T	WT	WT	WT	
	c.677C>T	WT	WT	WT	WT	WT	
c.12998A>C configuration	WT	WT	c.12998A>C	c.12998A>C	c.12998A>C	WT	
	WT	WT	WT	c.12998A>C	WT	WT	
Hypo fertile population	10.2%	24.3%	19.7%	9.2%	19.1%	17.2%	100%
(*N*)	(147)	(347)	(282)	(148)	(273)	(259)	(1430)
Endometriosis	21.5%	21.5%	16.9%	9.2%	21.5%	8.2%	100%
(*N*)	(34)	(34)	(26)	(17)	(34)	(13)	(158)
Likelihood ratio (Chi2)	**6.02**	2.59	1.9	0.07	0.03	**10.7**	
*p*	**0.0141**	0.1	0.17	0.79	0.86	**0.0011**	
Pearson (Chi2)	**6.7**	2.48	1.81	0.07	0.031	**9.14**	
*p*	**0.0097**	0.11	0.18	0.79	0.86	**0.0009**	
Fischer's (*p*)	**0.0091**	0.95	0.93	0.44	0.46	**0.0016**	

Bold values: Significant values and their degree of significance.

General hypofertile population.^15^ *N* number of patients.

SNP, single nucleotide polymorphism; WT, wild type.

**Table 2.** Odds Ratios for the Different Methylenetetrahydrofolate Reductase Combinations T677T/A1298A and Wild Type

	Odds ratio	Lower 95%	Upper 95%
c.677C>T; c.677C>T	1.74^*^	0.38	0.88
WT	0.42^*^	1.34	4.30

Bold^*^: significant values.

All others are not significant.

## Results

**FIG. 2.**
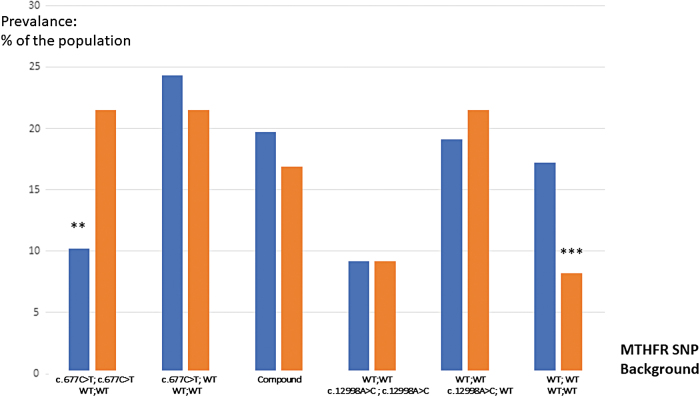
Prevalence of the different MTHFR combinations in our hypofertile population (1430) and the endometriosis subpopulation (158). Compound: compound heterozygote c.677C>T/c.1298A>C. *Red*: endometriosis; *Blue*: general hypofertile population. **0.01 significance; ***0.001 significance. WT, wild type.

The group of patients with endometriosis shows a distribution of SNPs that differs from that of the rest of the subfertile population (Tables 1 and 2; Fig. 2). The prevalence of the homozygous isoform is over-represented, with a 0.01 significance (adjusted Wald test: 0.01). Symmetrically, the percentage of WT patients affected in the endometriosis group is significantly lower, with a 0.001 significance (adjusted Wald test: 0.0001). The other combinations did not show any difference. The odd ratios show exactly the same features: the prevalence of WT is 2.4 times less and the prevalence of c.677C>T; c.677C>T (homozygous mutant) is 1.7 times higher.

Seventy-two patients have been co-treated with 5-MTHF, 7 are currently ongoing, waiting for pregnancy tests, 11 are waiting or had their frozen embryo transferred; the deliveries are available for 54 patients, with a success rate of 26/54 = 48.1%. Owing to the endometriosis-related obstetrical risks, only deliveries were taken into account.

## Discussion

Endometriosis is at the crossroads between oxidative stress and epigenetic/methylation disorders. The observations are in line with our previous observations.^24^ They reinforce significant observations linking oxidative stress with genetic polymorphisms in one-carbon metabolism.^4,6,24–26^ Estradiol-dependent oxidative stress, *via* estrogen receptors, affects methylation/epigenesis in reproduction. This is especially true if we consider that estradiol induces epigenetic regulation of gene expression.^8^ The folate and one-carbon cycles are at the epicenter of these fine-tuned regulatory biochemical pathways.

In term of pathophysiology, the influence of MTHFR mutations should not be overlooked in the treatment of endometriosis, especially in patients referred for significant duration of infertility.^15,27–29^ As mentioned earlier, the c.677C>T isoform carries the heaviest penalty: the compound heterozygote c.677C>T; c.1298A>C is by no means benign, as demonstrated by increased levels of circulating Hcy.^15^ However, the T677T MTHFR SNP cannot be regarded as an initiator of endometriosis, as WT patients can also be affected. It might be seen as an exacerbating factor, increasing the risk of oxidative stress and methylation anomalies.

Based on these observations, infertile endometriosis patients should be tested for the 677 isoforms principally; the c.1298A>C variant does not seem to be penalizing, in contrast to what is observed for the early embryo.^16,19^ The percentage of WT patients in a fertile population is between 40% and 55%.^20,27,29–31^ These two SNPs differ in their harmful effects on fertility: two studies^20,31^ investigating repeated miscarriages indicate an important negative influence of c.677C>T; c.677C> T (30.0% and 20.8%, respectively, in the two studies, vs. 7% and 4% in their fertile control groups).

In these two studies, the prevalence of the c.1298A>C variant is also significantly higher, with an increased incidence of multiple miscarriages in the carriers. In our group the c.1298A>C variant does not appear to have a major impact on the risk of endometriosis. Our patients carrying any form of these two MTHFR are always treated with MTHF: their folate metabolism naturally is impaired at the level of dihydrofolate reductase^32^ and then at the MTHFR stage: High doses of syntheic folic acid (FA) are not a suitable option, either in men or women.^27,28,33^

The resulting unmetabolized folic acid (UMFA), competing for FolR1 and SLC19A1, the receptor and transporter of all the folates, will affect epigenetic marking and gene regulation. Our preliminary results in this group of endometriosis patients co-treated with 5-MTHF are encouraging; a 48% ongoing pregnancy/delivery rate has been obtained without any obstetrical problems. Obstetrical complications have been currently described in relation with endometriosis,^34^ and MTHFR polymorphisms,^35,36^ our observations totally fit with this link. MTHFR SNPs and endometriosis have a cumulative effect on miscarriage.

For this reason, this rationale could be applied to all patients suffering with endometriosis. Determination of MTHFR SNPs could be proposed to these “difficult” patients after one or two miscarriages. Elevated estrogens generate oxidative stress induces placental/obstetrical pathologies.^37–39^ It is possible to conclude that all these observations should stimulate investigation on the prevalence of c.677C>T MTHFR SNP in infertile patients with endometriosis with increased obstetric risk and subsequently a possible decrease of this risk with 5-MTHF preventive administration, considering the low cost of the drug, its routinary use (in our hands) and prescription in the periconceptional period and its demonstrated harmlessness. These treatments have resulted in >250 pregnancies, mostly spontaneously,^27,30^ in MTHFR SNP carrier patients classified as having “idiopathic infertility.”

Carriers of the c.677C>T; c.677C>T isoform are routinely referred to a geneticist, on the basis of the additional pleiotropic health risks associated with increased levels of circulating homocysteine,^40–43^ unrelated to infertility; in particular psychiatric vascular disorders: MTHFR SNPs induce local/hidden methylation imbalances^16–18,39–43^ including the testis^33^ and the ovary.^44–46^ An impact on male germinal tissue in the embryo cannot be excluded, at the time when epigenetic resetting occurs.^47,48^

In conclusion, methylation is a major regulatory process throughout fertility: gametogenesis, as well as early and late embryogenesis and pregnancy in general. Our observations should stimulate investigation on the prevalence of c.677C>T; MTHFR SNP in infertile patients suffering endometriosis with increased obstetric risk, and subsequently a possible decrease of this risk with folate, especially 5-MTHF, preventive administration, and treatment during pregnancy.
